# ERRATUM: Measuring professional satisfaction and nursing workload among nursing staff at a Greek Coronary Care Unit

**DOI:** 10.1590/1980-220X-REEUSP-2024-ER03en

**Published:** 2024-09-30

In the article “Measuring professional satisfaction and nursing workload among nursing staff at a Greek Coronary Care Unit” with the DOI: https://doi.org/10.1590/S0080-6234201500000003, published by the journal: “Revista da Escola de Enfermagem da USP, volume 49 (spe) of 2015, p. 15–21, on page 21:

**Now read:**

## Financial Support

This study was financed in part by the Coordenação de Aperfeiçoamento de Pessoal de Nível Superior – Brasil (CAPES) – Finance Code 001.

